# Compositional data modeling of high-dimensional single cell RNA-seq (CoDA-hd): its advantages over commonly used normalization approaches

**DOI:** 10.1186/s12967-025-07157-z

**Published:** 2025-10-21

**Authors:** Jinghan Huang, Sheung Chi Phillip Yam, K. S. Leung, Minghua Deng, Nelson L. S. Tang

**Affiliations:** 1 Department of Chemical Pathology, Li Ka Shing Institute of Health Science, Faculty of Medicine, The Chinese University of Hong Kong, Hong Kong, SAR China; 2 Department of Statistics and Data Science, The Chinese University of Hong Kong, Hong Kong, SAR China; 3 Department of Computer Science and Engineering (CSE), The Chinese University of Hong Kong, Hong Kong, SAR China; 4 Cytomics Limited, Hong Kong Science Park, Hong Kong, SAR China; 5 School of Mathematical Sciences, Peking University, Beijing, China; 6 Hong Kong Branch of CAS Center for Excellence in Animal Evolution and Genetics and KIZ/CUHK Joint Laboratory of Bioresources and Molecular Research in Common Diseases, Hong Kong, SAR China; 7 Functional Genomics and Biostatistical Computing Laboratory, CUHK Shenzhen Research Institute, Shenzhen, China

**Keywords:** Single cell RNA-seq, Compositional data analysis, Centered-log-ratio, Data pre-processing, Normalization, Clustering, Trajectory inference

## Abstract

**Background:**

Compositional data analysis (CoDA) is an emerging statistical framework and has been extended to microbiome, bulk RNA-seq, and cell type proportions in single-cell RNA-seq (scRNA-seq), which typically has 50–200 components. Here, we explore the high-dimensional application of CoDA (CoDA-hd) and its various log-ratio (LR) transformations to raw count matrix of scRNA-seq which has over 20,000 components (e.g., protein coding genes). scRNA-seq matrices are typically sparse and high-dimensional. Common approaches of normalization such as log-normalization may lead to suspicious findings as previously shown for trajectory inference. Although RNA-seq is compositional data by nature, the geometry of CoDA in high-dimensional simplex is not compatible with most downstream analyses of scRNA-seq which are based on Euclidean space. In this study, we attempted to explore: (1) CoDA adaptability to scRNA-seq; (2) handling of zero data: prior-log-normalization, imputation or with specific count addition scheme; (3) transformation to Euclidean space and compatibility with downstream analyses.

**Results:**

Our results suggest that (1) the innovative count addition schemes (e.g., SGM) enable the application of CoDA to high dimensional sparse data (i.e., scRNA-seq); (2) log-normalized data could be transformed to CoDA LR representation; (3) CoDA LR transformations such as count-added centered-log-ratio (CLR) had some advantages in dimension reduction visualization, clustering, and trajectory inference in the tested real and simulated datasets. CLR provided more distinct and well-separated clusters in dimension reductions, improved the Slingshot trajectory inference, and eliminated the suspicious trajectory that was probably caused by the dropouts.

**Conclusions:**

We therefore conclude that CoDA may be a preferred scale-free model to handle scRNA-seq data for these downstream tasks. Additionally, an R package ‘CoDAhd’ was developed for conducting CoDA LR transformations for high dimensional scRNA-seq data. The code for implementing CoDA-hd, along with some example datasets, are available at https://github.com/GO3295/CoDAhd.

**Supplementary Information:**

The online version contains supplementary material available at 10.1186/s12967-025-07157-z.

## Background

Single cell RNA sequencing (scRNA-seq) has been widely used in biological and biomedical studies for exploring transcriptomic profiling and differences in various cell populations. In scRNA-seq data analysis, the first step is the normalization of raw counts of transcript abundances (TA) of genes so that total counts of TA are comparable across individual cells. The conventional normalization method is log-normalization as described and reviewed previously [1]. Subsequently, researchers developed SCTransform, which applies regularized negative binomial regression [2] to preprocess the data [3]. Typically, an scRNA-seq data matrix consists of ~ 20,000 genes × thousands to millions of cells captured for analysis and the gene expressions of every cell are represented as the counts in the matrix.

The dropout problem is an important and common artifact in scRNA-seq. Due to poorly understood experimental and technical factors, TA of some genes may randomly show zero counts. This phenomenon is not limited to low-expression genes, as housekeeping genes may also have zero TA counts in some cells. Although scRNA-seq data are compositional by nature, no previous attempts have been made to apply CoDA due to these difficulties (i.e., extreme high dimension and extremely sparse matrix). Most conventional normalization methods are not robust to such randomly missing data (dropouts). For example, PCA/UMAP performed with conventional methods may lead to suspicious findings due to the dropout problem (e.g., a controversial differentiation trajectory from plasmablasts to developing neutrophils using existing normalization methods, which was not plausible in biology [4–6]). Therefore, we are exploring the utility of alternative statistical models and data representation methods that are more robust to these properties and limitations of scRNA-seq data.

Here, we attempted the application of CoDA in the analysis of scRNA-seq. As an emerging school of statistical data analysis, the CoDA framework was first proposed by John Aitchison in the 1980s when it was applied to geochemical data and more recently to microbiome data (Fig. S1). The core difference between CoDA and the conventional scRNA-seq analysis is that CoDA explicitly treats data as log-ratios (LRs) between components, which can be correctly projected from compositional simplex geometry to Euclidean space after LR transformation. In contrast, the existing scRNA-seq analysis routine treats the TA data as real numbers in log space. Treating TA data as LRs rather than absolute values is designed to benefit from three intrinsic CoDA properties, which are scale invariance, sub-compositional coherence, and permutation invariance [7]. Scale invariance states that any scale (e.g., multiplied by a scale factor) of the original data will have no effect since compositional data only carries relative information. Sub-compositional coherence means that results obtained from a sub-composition (i.e., subset of data) will remain the same as in the composition. Permutation invariance is straightforward and states that the results do not depend on the order that the parts appear in a composition [8]. Another benefit of CoDA log-ratios is that they reduce data skewness and make the data more balanced for downstream analyses. These properties of CoDA make it a desirable model for scRNA-seq data.

**Fig. 1:**
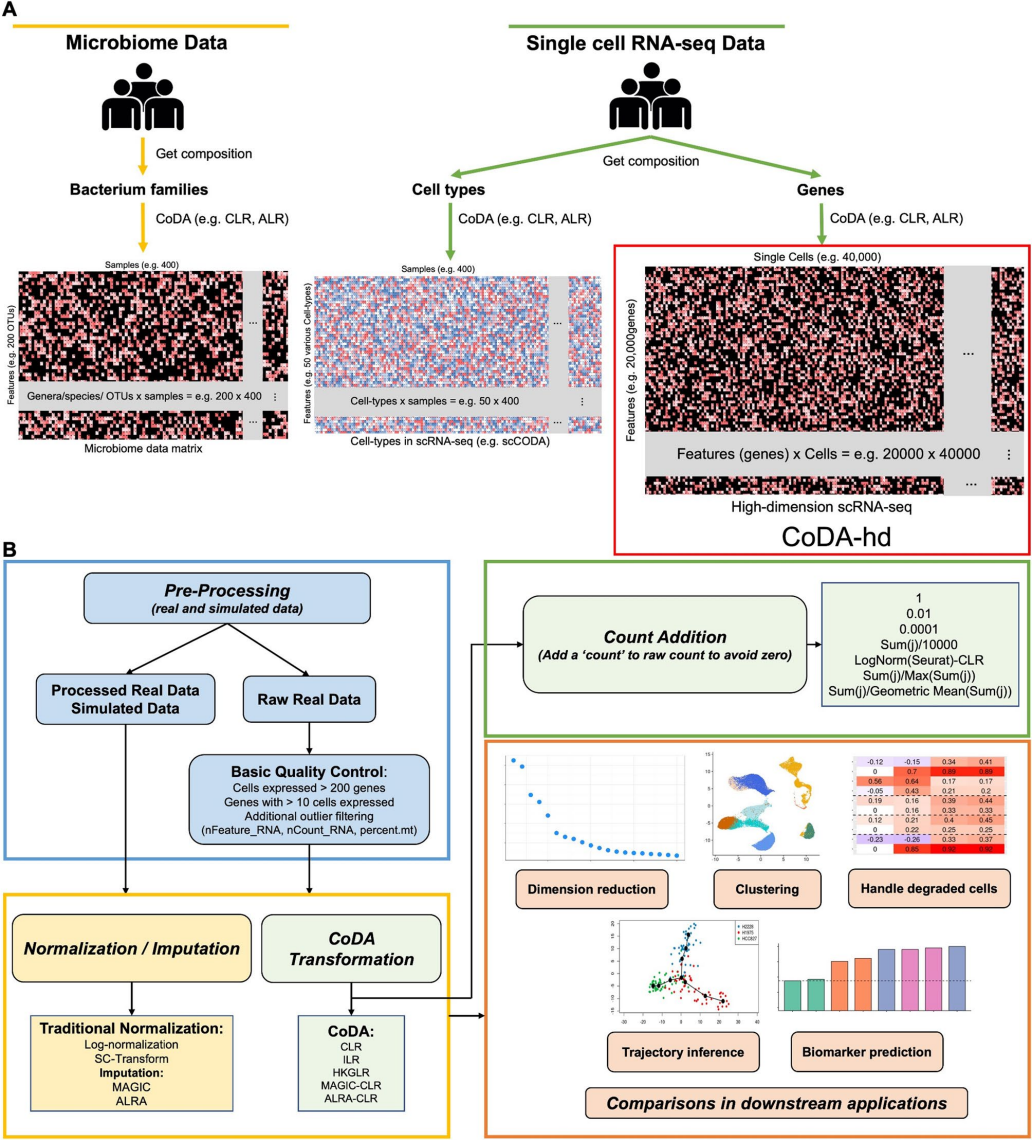
Concepts of compositional data analysis (CoDA) for different high-throughput sequencing data and the workflow in this study. **A** Current applications of compositional data analysis (CoDA) in different high-throughput sequencing data are illustrated. At present, CoDA only confined to hundreds of components or features. For example, there are hundreds of OTUs in Microbiome data and up to fifty cell type in cell count proportion data. Our study extends its use in much higher dimensional scRNA-seq data (up to tens of thousands of components) where each gene is treated as one component. **B** The workflow of analysis of twenty-nine real datasets (including a collection of 15 ‘gold standard’ trajectory datasets) and four simulated datasets (i.e., Splatter and SplatPop) which are pre-processed with quality control, normalization/imputation, or CoDA transformations. Performances of various CoDA transformations are evaluated by commonly used algorithms for downstream applications

Theoretically, CoDA has great potential to be used in high-throughput sequencing (HTS) data. Its application to microbiome data and bulk RNA sequencing data have been demonstrated. For microbiome data, bacterium proportions (Fig. 1A, Bacterium) in a stool sample are compositional in nature and CoDA is readily applied to them. It is also proposed that CoDA could be used to analyze bulk RNA-seq due to the compositional nature of the data [8, 9]. Bulk RNA-seq sums the TA of all cells in one sample and has been frequently used to explore the TA difference between different conditions. The bulk expression matrix is much smaller than the scRNA-seq matrix. In fact, HTS bulk or single cell RNA-seq naturally generate relative information of feature abundance and thus has an upper limit of total number of reads. This upper bound arises because sequencers can only process a fixed number of nucleotide fragments. Therefore, a theoretical competitive situation may occur where the number of one transcript increases will actually decrease the number of all other transcripts observed [8]. As another example, CoDA is used in metagenomics datasets for various microbiomes [10–12]. Besides, researchers have developed several R CoDA packages to perform the gene differential abundance analysis, conduct simulation study or study cell type proportions (Fig. 1A, scRNA-seq cell types) [12–14], for small data matrix of size up to 1000 $\times$ 1000. Various benchmarks for comparing other methods with CoDA were also conducted [8, 10, 15–17]. In CITE-seq, the size of the matrix of cell surface protein expression is usually ~ 100 proteins $\times$ thousands to millions cells, and the data are often handled by CoDA centered-log-ratio (CLR) transformation [18]. Compared to these existing CoDA applications in biology, treating genes as components in scRNA-seq poses major challenges, e.g., thousands to millions of cells are handled, and result in a 1000-fold increase in columns for a typical CoDA matrix. As the sizes of the data matrices range from 20,000 genes $\times$ thousands to millions of cells, we applied CoDA and named it CoDA-high dimensional (CoDA-hd) and explore the adaptability and output of such applications (Fig. 1A, scRNA-seq Genes).

**Fig. 2:**
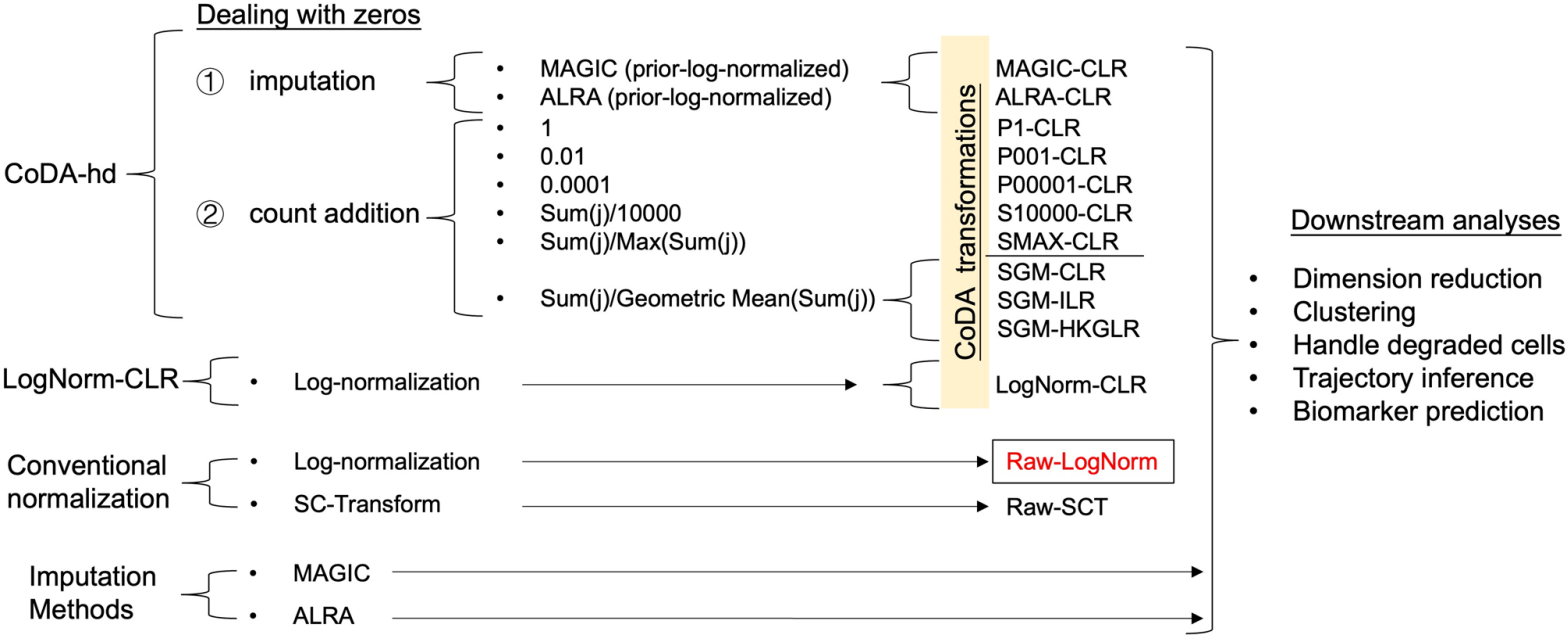
Details of the strategies of comparison among CoDA, conventional normalization and imputation methods, and overview of workflows in this study. Details of the framework in this study are presented. In CoDA-hd framework, two categories of methods (i.e., imputation and count addition) are applied to deal with zeros in scRNA-seq datasets before CoDA transformation by various log-ratio representations. In addition, conventional normalized data and imputed data are also forwarded for LR transformation. The effects with or without LR transformation were evaluated by five categories of downstream analyses (including dimension reduction, cell clustering, interference due to degraded cell, trajectory analysis and biomarker identification). Raw-LogNorm representing the typical Log-Normal transformation routine applied to scRNA-seq data by Seurat package is used as the baseline in all subsequent comparisons for CoDA performance

scRNA-seq data are highly sparse: with a large proportion of zeros, it can lead to false discovery or ambiguous conclusions, as noted [4]. This study also explores and compares strategies for handling zeros, which is the key challenge in CoDA transformations (Fig. 1B and 2). Previous work has documented that CoDA processing faces significant hurdles with low-count matrices, posing a challenge for their analysis [17]. Additionally, it was demonstrated that applying a transformation for conceptual reasons does not necessarily translate into better downstream analysis results [3].

Since the application of CoDA in scRNA-seq has not been explored, here we assess if CoDA as a normalization approach, which treats the TA counts of every single cell as compositional, can be more robust and perform better in various scRNA-seq downstream applications (Figs. 1B, 2 and Table S1). Motivated by the potential limitations of traditional log-normalization, we apply CoDA for both real and simulated scRNA-seq datasets and compare its performance with other methods.

## Methods

### Conventional normalization methods for scRNA-seq data

Existing normalization methods ignored the compositional nature of scRNA-seq data and treat the dataset in Euclidean space. Moreover, many data repositories only provide prior-normalized data. It has not been explored if such prior-normalized data matrices are compatible with subsequent CoDA LR transformation. The two common normalization methods (Table S2, Group A) are (1) log-normalization, by the function NormalizeData() and (2) SC-Transformation (regularized negative binomial regression) using SCTransform(). Both log-normalization and SC-Transform were implemented in Seurat [2]. Theoretically, data after log-normalization (default normalization in Seurat) could be converted into CoDA LR representation, as we demonstrated below.

### Transform scRNA-seq data to CoDA log-ratio (LR) representations

In CoDA, the true absolute counts are assumed to be unobserved, and only the relative proportions between parts are considered valid. ‘Part’ is the terminology used in CoDA for components or variables (genes in the context of scRNA-seq). The principal difference between CoDA and existing analysis methods is that data are represented as log-ratios of parts in CoDA, while conventional non-CoDA methods treated the data as real numbers. The key hurdle of using CoDA to analyze scRNA-seq data is its sparsity. LR is required to transform the original CoDA data present in a simplex geometry compatible with most downstream analyses designated in Euclidean geometry [15]. Therefore, zero counts are inherently incompatible with CoDA. Here, we explored two categories of methods to replace zero, (1) count addition and (2) imputation of missing data. A new scheme of count addition proposed here may be the most optimal method applicable to scRNA-seq. Furthermore, we compared our proposed count addition scheme against conventional normalization methods (log-normalization, SC-Transform) and imputation methods (MAGIC, ALRA) across multiple scRNA-seq datasets.

1. Handling of sparse matrix by count addition and CoDA LR transformations

In CoDA, each component (gene) represents a certain part taking up a particular percentage component of the whole specimen, where all measured parts sum to unity (e.g., 100%). For TA count data, the TA of all genes sums to 1 for each cell, with each gene’s abundance expressed as a proportion of the total transcripts in that cell. These proportional abundances are then transformed using various CoDA LR methods. To avoid zeros in LR process, we applied various commonly used count addition and introduced a novel count addition scheme that could be more applicable to single cell HD matrices. We applied these methods to the raw counts and evaluated their performance (Figs. 1B and 2).

CoDA LR transformation is applied after performing the following different schemes of count-addition to the sparse (zero-inflated) raw scRNA-seq data. This is also the widely used method in typical CoDA of standard data matrix of lower dimension.

- Count addition by a constant value (constant value addition). Previous studies reported several ways to handle zeros for CoDA such as the addition of half minimum value to only those zeros (i.e., a fixed assumed value for counts below the detect limit) [7]. However, these methods did not work well for scRNA-seq data as shown in results. Specifically, a fixed constant of 1, 0.01, or 0.0001 was added to all raw counts in the whole matrix before closure. And the LR transformations (e.g., CLR) were performed on the proportions after closure:
  $$ x_{{fixed,i,j}}^{\prime } = x_{{i,j}} + 1\;or\;0.01\;or\;0.0001 $$
  $$ CLR(x_{{fixed,i,j}} ) = \log \left( {\frac{{x_{{fixed,i,j}}^{\prime } }}{{G(x_{{fixed,j}}^{\prime } )}}} \right) $$
  where ${x}_{i,j}$ is the raw count of the $i$ gene and $j$ cell. $G({x}_{j}{\prime})$ is the geometric mean of all genes of cell $j$.

We discovered that the constant value addition method resulted in poor performance (see results). Therefore, other count addition schemes were explored.

(b) Count addition by a fraction with constant scaling denominator. Scaling to the same total count per cell (each column) in the experiment is a common practice in normalization by scaling, e.g., log-normalization (LogNorm). Here, the pseudocount (terminology in the normalization literature) addition takes place after transforming to the proportions and scaling. The scaling factor is usually defined as 10,000 (also used in Seurat by default):
  $$ \begin{aligned} x_{{LogNorm,i,j}} & = {\text{log}}\left( {\frac{{x_{{i,j}} \times 10000}}{{\sum\limits_{{i = 1 \ldots D}} {x_{{i,j}} } }} + 1} \right) \\ & = {\text{log}}\left( {\frac{{x_{{i,j}} \times 10000 + \sum\limits_{{i = 1 \ldots D}} {x_{{i,j}} } }}{{\sum\limits_{{i = 1 \ldots D}} {x_{{i,j}} } }}} \right) \\ \end{aligned} $$

Therefore, for CoDA CLR implementation here, a value of S/10000 (S = sum of the counts per cell) was used as the ‘count’ added to the raw counts of each cell (each column):

$$ \begin{aligned} x_{{S/10000,i,j}}^{\prime } & = x_{{i,j}} + \frac{{\sum\limits_{{i = 1 \ldots D}} {x_{{i,j}} } }}{{10000}} \\ & \quad = \frac{{10000x_{{i,j}} + \sum\limits_{{i = 1 \ldots D}} {x_{{i,j}} } }}{{10000}} \\ \end{aligned} $$

$$ CLR(x_{{S/10000,i,j}} ) = \log \left( {\frac{{x_{{S/10000,i,j}}^{\prime } }}{{G(x_{{S/10000,j}}^{\prime } )}}} \right) $$

It is interesting to note that this step is equivalent as performing LR transformation for prior-log-normalized data (see below).

(c) Count addition by a fraction with both variable numerator and denominator. Then, we proposed a novel category of count addition. The scaling factor of 10,000 was selected for optimal performance with scRNA-seq data matrix of the current experimental setup of sequencing depth. With increasing sequencing power, the prevailing sequencing depth will increase for future scRNA-seq experiments, therefore both numerator and the denominator should be experiment or results dependent. Thus, we developed the following two count addition schemes characterized by having both variable numerator and denominator:
  1. S_j_/Max(S_j_) (S = sum of the counts per cell): Add S_j_/Max(S_j_) to per cell j (every column) raw count, and has a maximum addition of 1.
    $$ \begin{aligned} x_{{S/{\text{Max}}(S),i,j}}^{\prime } & = x_{{i,j}} + \frac{{\sum\limits_{{i = 1 \ldots D}} {x_{{i,j}} } }}{{{\text{Max}}\left( {\sum\limits_{{i = 1 \ldots D}} {x_{{i,j}} } } \right)}} \\ & \quad = \frac{{{\text{Max}}\left( {\sum\limits_{{i = 1 \ldots D}} {x_{{i,j}} } } \right)x_{{i,j}} + \sum\limits_{{i = 1 \ldots D}} {x_{{i,j}} } }}{{{\text{Max}}\left( {\sum\limits_{{i = 1 \ldots D}} {x_{{i,j}} } } \right)}} \\ \end{aligned} $$
    $$ CLR(x_{{S/Max(S),i,j}} ) = \log \left( {\frac{{x_{{S/Max(S),j}}^{\prime } }}{{G\left( {x_{{S/Max(S),j}}^{\prime } } \right)}}} \right) $$
  2. S_j_/GM(S_j_) (S = sum of the counts per cell, GM = geometric mean of per cell (every column) total counts across cells):
    $$ \begin{aligned} x_{{S/GM,i,j}}^{\prime } & = x_{{i,j}} + \frac{{\sum\limits_{{i = 1 \ldots D}} {x_{{i,j}} } }}{{{\text{G}}\left( {\sum\limits_{{i = 1 \ldots D}} {x_{{i,j}} } } \right)}} \\ & \quad = \frac{{{\text{G}}\left( {\sum\limits_{{i = 1 \ldots D}} {x_{{i,j}} } } \right)x_{{i,j}} + \sum\limits_{{i = 1 \ldots D}} {x_{{i,j}} } }}{{{\text{G}}\left( {\sum\limits_{{i = 1 \ldots D}} {x_{{i,j}} } } \right)}} \\ \end{aligned} $$
    $$ CLR(x_{{S/GM,i,j}} ) = \log \left( {\frac{{x_{{S/GM,i,j}}^{\prime } }}{{G(x_{{S/GM,j}}^{\prime } )}}} \right) $$
    where $\text{G}(\sum_{i=1\dots D}{x}_{i,j})$ is the geometric mean of per cell (every column) total counts across cells and $G(x_{j}^{\prime } )$ is the geometric mean of all genes of cell $j$.

2. Transferability of log-normalized data to CoDA LR transformations

As many datasets have been pre-normalized by conventional log-normalization with scaling, we explored and showed here that such normalizations could be transferable to CoDA by various LR transformations. We introduced an approach to implement count fraction addition with a constant denominator of 10,000 (method b above). CoDA LR transformation based on log-normalization (CLR (LogNorm)) was conducted in log-space, and the main point is that the count of 1 was added to the ‘normalized’ count (proportions scaled to 10,000), followed by logarithm. Specifically for CLR, data was first log-normalized and then subtracted by the arithmetic means of ‘selected genes’ (all genes for CLR):

$$ \begin{aligned} CLR(x_{j} ) & = \log \left( {\frac{{x_{{i = 1 \ldots D,j}}^{\prime } }}{{G\left( {x_{j}^{\prime } } \right)}}} \right) \\ & \quad = \log \left( {x_{{i = 1 \ldots D,j}}^{\prime } } \right) - \frac{1}{D}\sum\limits_{{i = 1 \ldots D}} {\log } (x_{{i,j}}^{\prime } ) \\ \end{aligned} $$

$$ \begin{aligned} x_{{i,j}}^{\prime } {\text{ }} & = \frac{{x_{{i,j}} }}{{\sum\limits_{{i = 1 \ldots D}} {x_{{i,j}} } }} \times 10000 + 1 \\ & \quad = \frac{{10000x_{{i,j}} + \sum\limits_{{i = 1 \ldots D}} {x_{{i,j}} } }}{{\sum\limits_{{i = 1 \ldots D}} {x_{{i,j}} } }} \\ \end{aligned} $$

Note: term $\left(\frac{{x}_{i,j}}{\sum_{i=1\dots D}{x}_{i,j}}\times 10000\right)$ will not affect log-ratios as they are scale invariant.

Then

$$ \begin{aligned} CLR(x_{j} ) & = \log \left( {10000x_{{i,j}} + \sum\limits_{{i = 1 \ldots D}} {x_{{i,j}} } } \right) - \log \left( {\sum\limits_{{i = 1 \ldots D}} {x_{{i,j}} } } \right) \\ & \quad - \frac{1}{D}\sum\limits_{{i = 1 \ldots D}} {\log } \left( {\frac{{10000x_{{i,j}} + \sum\limits_{{i = 1 \ldots D}} {x_{{i,j}} } }}{{\sum\limits_{{i = 1 \ldots D}} {x_{{i,j}} } }}} \right) \\ & = \log \left( {10000x_{{i,j}} + \sum\limits_{{i = 1 \ldots D}} {x_{{i,j}} } } \right) \\ & \quad - \log \left( {\sum\limits_{{i = 1 \ldots D}} {x_{{i,j}} } } \right) \\ & \quad - \frac{1}{D}\sum\limits_{{i = 1 \ldots D}} {\log } \left( {10000x_{{i,j}} + \sum\limits_{{i = 1 \ldots D}} {x_{{i,j}} } } \right) \\ & \quad + \frac{1}{D}\sum\limits_{{i = 1 \ldots D}} {\log } \left( {\sum\limits_{{i = 1 \ldots D}} {x_{{i,j}} } } \right) \\ & = \log \left( {10000x_{{i,j}} + \sum\limits_{{i = 1 \ldots D}} {x_{{i,j}} } } \right) \\ & \quad - \frac{1}{D}\sum\limits_{{i = 1 \ldots D}} {\log } \left( {10000x_{{i,j}} + \sum\limits_{{i = 1 \ldots D}} {x_{{i,j}} } } \right) \\ \end{aligned} $$

where ${x}_{i,j}$ is the raw count of the $i$ gene and $j$ cell. Term $\sum_{i=1\dots D}{x}_{i,j}$ served as the ‘count addition’ that differs among cells.

Note that the above step is a special case for sparse scRNA-seq data when performing CoDA and is equivalent as centering the data within each cell (every column) by selected features (e.g., all features for CLR) in log-space. Therefore, the inflated-zero-effect will be mitigated as well. Theoretically, log-ratios in any CoDA data could still be zero if adding a count of 1 when taking the logarithm of zero. However, this is nearly impossible in scRNA-seq data since most of the genes contains zeros across cells and the calculation of the geometric means requires no zeros.

3. Various LR transformations of CoDA evaluated

After various scheme of management of zero counts, the data matrix is ready for CoDA using various kinds of LRs (e.g., CLR, IQLR, HKGLR and ILR) with various advantages and limitations. CLR centered the data by geometric means, while IQLR uses quartile values. HKGLR uses housekeeping gene expressions as reference. ILR is the most robust in CoDA with invariant and isometric properties. Various LRs were then subjected to dimension reduction, clustering and other scRNA-seq downstream tasks for evaluations and were also compared with results generated from typical Seurat workflow using LogNorm or SC-Transform (Fig. 2). The details of the various LR formulations were shown in Table S2, Group CoDA. Note that various LRs use different formulation for the denominator which will affect the output and sometimes makes it difficult for direct interpretation (e.g., ILR) [9].

1. The most common LR expression of CoDA is the Centered-Log-Ratio (CLR) which takes the geometric mean of all features (genes) as denominators, defined as:
  $${CLR(x}_{j})=\text{log}(\frac{{x}_{i=1\dots D,j}}{G({x}_{j})})$$
  where $G({x}_{j})$ is the geometric mean of the D features (genes) of sample/cell ${x}_{j}$.
2. A more complicated LR transformation, Isometric-Log-Ratio (ILR), is defined as:
  $$ ILR(x_{j} ) = \sqrt {\frac{{D - i}}{{D - i + 1}}} {\text{log}}\left( {\frac{{x_{{i = 1 \ldots D,j}} }}{{\sqrt[{D - i}]{{\prod _{{k = i + 1}}^{D} x_{{k,j}} }}}}} \right) $$
3. A knowledge-based transformation Housekeeping-Gene-Log-Ratio (HKGLR): takes the geometric mean of five housekeeping genes (*SDHA*, *ACTB*, *UBC*, *YWHAZ*, *GAPDH*; or other reasonable genes) as the denominator:
  $$ HKGLR(x_{j} ) = {\text{log}}\left( {\frac{{x_{{i = 1 \ldots D,j}} }}{{G_{j} (x_{{j,HKG}} )}}} \right) $$

Usually, housekeeping genes have stable expression.

Notably, in addition to the above CoDA LR transformations, some other LR concepts are also well described in the CoDA field or have been proposed previously [7, 12, 19], e.g., IQLR, LVHA, mdCLR or group-based LR. We evaluated these transformations as well (data not shown) but found no improvement or worse performance than the most representative transformation CLR, and thus, only the well-known CLR, ILR, and HKGLR were included in the result part for simplicity.

4. Imputation for zero counts

Two algorithms (i.e., MAGIC & ALRA) for imputation of zero counts in scRNA-seq data matrix (Table S2, Group B) were also evaluated and compared with CoDA results. Their outputs were CLR-transformed (based on LogNorm) for downstream evaluations as well.

### Data collection and pre-processing

Datasets used in this study were collected from the Gene Expression Omnibus (GEO) and other public resources, as described in the original studies [20–28]. Most of these datasets had been used for algorithm evaluations or benchmark analyses and were considered high quality [20, 25, 28, 29]. Simulated scRNA-seq datasets were generated using two well-established R packages: Splatter [30] and SplatPop [31]. Details of these datasets are shown in Table S3. Processed datasets were obtained from previous studies. For the four simulated datasets, we investigated whether CoDA LR transformations could better reflect the ground truth. We also evaluated the performance of various normalizations/LR transformations on dimension reduction and clustering using simulated datasets without dropouts (ground truth). For raw datasets lacking prior quality control, we applied the following filtering criteria to remove: (1) Cells expressing fewer than 200 genes; (2) outlier cells based on number of genes expressed (nFeature_RNA), number of total counts (nCount_RNA), and percentage of mitochondrial gene expression (percent.mt); (3) Genes expressed in less than 10 cells.

### Evaluation methods: overview

We evaluated the performance of different normalization methods and CoDA LR transformations across multiple downstream tasks. Since log-normalization (LogNorm) is the most widely used method in scRNA-seq analysis, we used it as the baseline for comparisons. Specifically, to compare the performance metrics across methods, we subtracted the LogNorm results from those of other approaches and visualized the differences using box plots, bar plots, and heatmaps. Note that extreme values were thresholded (cutoff) in some analyses for clearer visualization.

### Dimension reduction and clustering

1. Using partial SVD to replace LRA (R-easyCODA) in performing CoDA dimension reduction and clustering

**Fig. 3:**
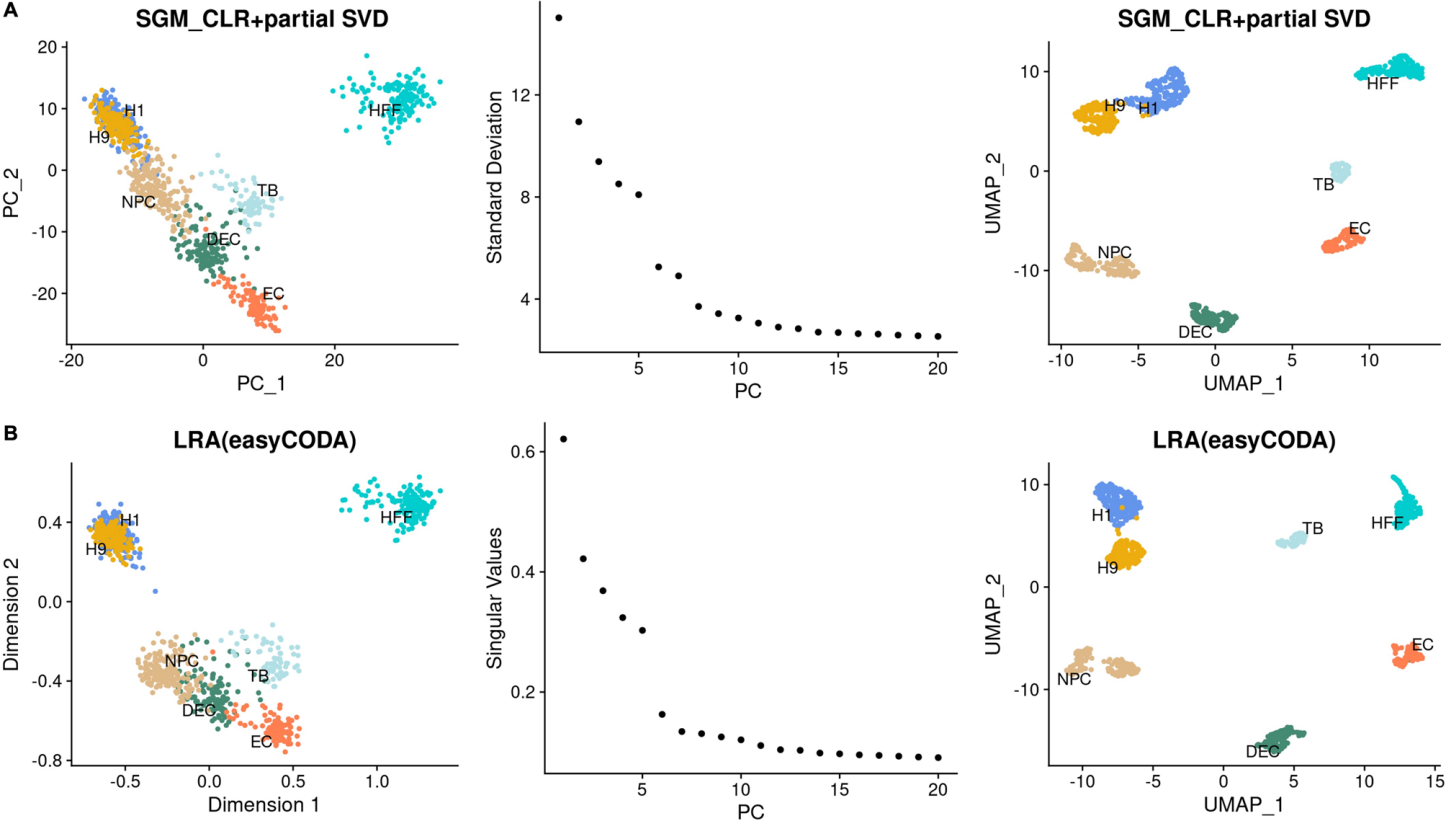
Comparison of the first 2-D PCA & UMAP plots and elbow plots of dimension reduction algorithms in CoDA–The new CLR + partial SVD vs. LRA (typical CoDA dimension reduction algorithm in the easyCODA R-package) using the GSE75748-CellType dataset. To speed up the dimension reduction process for the high dimensional scRNA-seq data, we perform partial SVD on the CLR (SGM) transformed data and compare the resulted PCs with the standard dimension reduction performed by the LRA command in the easyCODA R package for CoDA data (top 3000 features × 997 cells). The first 2-D PCA & UMAP plots and the elbow plots for the dataset GSE75748-CellType for each of the methods are shown for comparison. Overall, the clusters of results are similar between the two algorithms. Therefore, the fast CLR + partial SVD is used in subsequent analysis. **A** Results of the partial SVD on CLR (SGM) transformed data. **B** Results of the LRA (easyCODA) based on CLR (SGM)

CoDA enables unbiased dimension reduction by principal component analysis (PCA) and biplots analysis on log-ratio data [7]. Specifically, the log-ratio analysis (LRA; implemented in easyCODA R Package) is a special case of PCA that applied to the CLR. However, it is not designed for high-dimension data like scRNA-seq matrix, and thus it took a very long time (virtually impractical) to run in ‘easyCODA’. Here, we showed that our CoDA-hd ‘CLR + partial SVD’ process using fast truncated Singular Value Decomposition (SVD) could resulted in an approximation solution–the resulted PCA had similar patterns as those generated by typical CoDA procedure (LRA function) by the ‘easyCODA’ package (Figs. 3 and S2). The partial SVD was conducted using the package ‘irlba’, which applies implicitly-restarted Lanczos methods for fast truncated SVD of sparse and dense matrices [32]. Therefore, it provides much better scalability than LRA in ‘easyCODA’.

2. Quantitatively evaluate the clustering performance of different normalizations and CoDA LR transformations

We adopted four evaluation metrics, Entropy of accuracy (${H}_{acc}$), Entropy of purity (${H}_{pur}$), Adjusted Rand Index (ARI) and Normalized mutual information (NMI)[25, 29] with K-means Clustering and Louvain Clustering algorithms on multiple labelled (i.e., with ground truth cell type label) datasets. For clustering, the number of clusters was set to be the number of known cell type labels in each dataset. The top 3000 variable genes and the top 10 PCs were used for the cell clustering. The detailed implementations of the clustering algorithms were described previously [25].

3. Metrics to access clustering concordance with the ground truth

${H}_{acc}$ Evaluates the difference of the true groups within each predicted cluster, defined as:

$${H}_{acc}=-\frac{{\sum }_{i=1}^{M}{\sum }_{j=1}^{{N}_{i}}{p}_{i}({x}_{j})\text{log}({p}_{i}({x}_{j}))}{M}$$

$$0\le {H}_{acc}\le \text{log}(M)$$

where $M$ is the number of predicted clusters; ${N}_{i}$ is the number of true groups in the ${i}^{th}$ predicted cluster; ${x}_{j}$ are cells in the ${j}^{th}$ true group; and ${p}_{i}({x}_{j})$ are the proportions of cells in the ${j}^{th}$ true group relative to the total number of cells in the ${i}^{th}$ predicted cluster.

${H}_{pur}$ evaluates the difference of the predicted clusters within each true group, defined as:

$${H}_{pur}=-\frac{{\sum }_{i=1}^{N}{\sum }_{j=1}^{{M}_{i}}{p}_{i}({x}_{j})\text{log}({p}_{i}({x}_{j}))}{N}$$

$$0\le {H}_{pur}\le \text{log}(N)$$

where $N$ is the number of true group; ${M}_{i}$ is the number of predicted clusters in the ${i}^{th}$ true group; ${x}_{j}$ are cells in the ${j}^{th}$ predicted cluster; and ${p}_{i}({x}_{j})$ are the proportions of cells in the ${j}^{th}$ predicted cluster relative to the total number of cells in the ${i}^{th}$ true group. Smaller ${H}_{acc}$ and ${H}_{pur}$ indicates better clustering performance [25].

Adjusted Rand index (ARI) that evaluates the similarities between two data distributions [29] was calculated using adjustedRandIndex() function in mclust package [33], defined as:

$$ ARI = \frac{{\sum _{{ij}} \left( \begin{gathered} n_{{ij}} \hfill \\ 2 \hfill \\ \end{gathered} \right) - \left[ {\sum _{i} \left( \begin{gathered} a_{i} \hfill \\ 2 \hfill \\ \end{gathered} \right)\sum _{j} \left( \begin{gathered} b_{j} \hfill \\ 2 \hfill \\ \end{gathered} \right)} \right]/\left( \begin{gathered} n \hfill \\ 2 \hfill \\ \end{gathered} \right)}}{{\frac{1}{2}\left[ {\sum _{i} \left( \begin{gathered} a_{i} \hfill \\ 2 \hfill \\ \end{gathered} \right) + \sum _{j} \left( \begin{gathered} b_{j} \hfill \\ 2 \hfill \\ \end{gathered} \right)} \right] - \left[ {\sum _{i} \left( \begin{gathered} a_{i} \hfill \\ 2 \hfill \\ \end{gathered} \right)\sum _{j} \left( \begin{gathered} b_{j} \hfill \\ 2 \hfill \\ \end{gathered} \right)} \right]/\left( \begin{gathered} n \hfill \\ 2 \hfill \\ \end{gathered} \right)}} $$

$${n}_{ij}=\sum_{k,g}I({u}_{k}=i)I({v}_{g}=j)$$

$${a}_{i}=\sum_{k}I({u}_{k}=i)$$

$${b}_{j}=\sum_{g}I({v}_{g}=j)$$

$$ I\left( {x = y} \right) = \left\{ {\begin{array}{*{20}l} {1,} \hfill & {x = y} \hfill \\ {0,} \hfill & {otherwise} \hfill \\ \end{array} } \right. $$

where $i$ and $j$ enumerate the $k$ clusters; ${\{{u}_{i}\}}_{i}^{m}$ is the predicted cluster label; ${\{{v}_{j}\}}_{j}^{m}$ is the true group label.

Normalized mutual information (NMI) that measures the correlation between two random variables [29] is calculated using NMI() function from aricode package [34], defined as:

$$ NMI = 2\frac{{I\left( {G,\hat{G}} \right)}}{{H\left( G \right) + H\left( {\hat{G}} \right)}} $$

$$ I\left( {G,\hat{G}} \right) = \mathop \sum \limits_{{a \in G,b \in \hat{G}}} p\left( {a,b} \right)\ln \frac{{p\left( {a,b} \right)}}{p\left( a \right)p\left( b \right)} $$

$$ H\left( G \right) = \mathop \sum \limits_{a \in G} p\left( a \right)\ln p\left( a \right) $$

where $G$ is the true group, $\widehat{G}$ is the predicted cluster. $p\left(a\right)$, $p\left(b\right)$ and $p\left(a,b\right)$ are the probabilities that the cell belongs to cluster a, cluster b and both, respectively. For both ARI and NMI, higher values indicate better clustering performance.

To compare the above metrics among different methods, we first subtracted all the ARI and NMI values by the results of the log-normalized data (vice versa for ${H}_{acc}$ and ${H}_{pur}$ for better visualization) and then took the median values of the four real or simulated datasets. The heatmaps were generated for both median aggregates and individual dataset (note that the extreme values have been limited to a specific cutoff for better visualization). Next, 2-D PCA or UMAP plots were generated for each dataset to evaluate the performance of different methods on dimension reductions. The first 10 PCs were used to generate the UMAP.

For the four simulated datasets, we further evaluated the performances of various normalizations/LR transformations using the ground truth (no dropout) simulated datasets.

### Effect of zero-inflation on dimension reduction

Low-quality cells (e.g., degraded cells or cell debris) are common in scRNA-seq datasets. To simulate the effect of degraded cells, we spiked in 10% zero-inflated cells into high-quality datasets (CellBench-10X-5CL and GSE75748). For example, 10% of the H1975 cells (a cell line originated from a lung tumor) were randomly selected and copied to simulate low-quality cells. In each copied cell, 60% of the genes (~ 6000 genes) were randomly assigned to zero. Thus, the new dataset has 10% more degraded H1975 cells with at least 70% features set to zero (i.e., zero-inflated vectors). The data were then processed by various normalization methods and CoDA LR transformations, followed by evaluations via PCA and UMAP. Additionally, clustering performance was evaluated based on the subset cell of H1975 and H1975-zero. Similarly, 40% of the H1 cells from dataset GSE75748-CellType were copied and simulated as described above, with subsequent evaluation of the H1 and H9 cell subsets.

### Trajectory inference

To evaluate the performance of different methods on trajectory inference, we applied Slingshot [35], DPT [36], Monocle2 and Monocle3 [37] on twenty-two real datasets with known cell-state/time labels (Table S3). Spearman correlation coefficients (SCC) were calculated between the predicted pseudotime and the ground truth time labels. Another metric Pseudo-temporal ordering score (POS) which measures cell orders were calculated using orderscore() in TSCAN [38] package. Due to space limitation and scalability, fifteen ‘gold standard’ datasets from Saelens et al. [28] were not used in MAGIC and ALRA imputation.

In Slingshot, the starting point of the trajectory was set based on the known starting time and other parameters were set to default. For trajectory with multiple branches, the predicted branches and the real branches were matched by their largest overlaps of cells. Specifically, for datasets with three cell types (A, B, and C where A will differentiate to B and C) with even number, ideally predicted branch 1 would have 100% overlap rate with A-to-B known branch while only have 50% overlap rate with A-to-C known branch (vice versa for predicted branch 2). Then the true overlap rates (i.e., largest overlaps between predicted branches and real branches, as matched branches), trajectory false rates (average of percentage of cells in selected real branch that appear in other predicted branches), incorrect cell type proportions (percentage of cells in matched predicted branch that did not belong to matched real branch) were calculated. 2-D PCA plots were generated for visualizing Slingshot results.

In DPT, same procedures were applied and the predicted branches by DPT were further evaluated with clustering metrics ${H}_{acc}$, ${H}_{pur}$ ARI, and NMI (as described in Clustering). 2-D diffusion map plots were generated for visualizing trajectories. In Monocle2 & 3, same procedures were applied.

### Biomarker predictive performance

Ratio-based biomarkers have been reported to perform well in classifying or predicting disease status [39, 40]. Indeed, researchers have used gene abundance ratios in qPCR data to study expression differences between conditions. In this study, we evaluated the performance of different transformations in cell type biomarker identification using three real scRNA-seq datasets with matched bulk (GSE75748, GSE81861, CellBench-10X-5CL) and three simulated datasets 1–3 (Table S3). The top 10 differentially expressed genes (DEGs) between pairwise cell types—identified in bulk data or ground-truth DEGs in simulated datasets—were used for evaluation in scRNA-seq data. Predictive performance was evaluated by calculating the area under the receiver operating characteristic curve (AUROC) for each selected gene in each comparison. We chose AUC here because it is a robust metric without any statistical assumption, unlike other differential expression analysis methods that rely on specific statistical basis/assumptions which may be incompatible with compositional or other data types. The AUC values were compared among different normalizations/LR transformations. We also calculated the percentage of marker genes with AUC values above predefined cutoffs. For simulated datasets, 50 known non-marker genes (ground-truth non-DEGs) between pairwise cell types were randomly selected out and their AUC were computed to estimate potential false positive rates.

### Other statistical analysis

For each of the comparisons with enough data points, Wilcoxon rank sum tests were performed between the results of the selected methods and the conventional log-normalization. The significance levels were shown in each box plot with *, **, *** represents ‘*P* < 0.05’, ‘*P* < 0.01’ and ‘*P* < 0.005’ respectively, and ‘#’ stands for not-significant-difference (‘*P* < 0.1’).

### Running time and memory usage

We used four datasets (10,000 $\times$ 1500, 10,000 $\times$ 5000, 10,000 $\times$ 10,000 and 10,000 $\times$ 50,000 matrix) to evaluate the time spent and memory usage of different methods (Log-normalization, SC-Transform and CLR). Additionally, we compared the running time and memory usage between LRA-easyCODA and CLR-partial-SVD. The top 3000 variable features were used in LRA and partial-SVD. To obtain the maximum memory usage, we used the peakRAM() function from the ‘peakRAM’ package.

## Results

We first evaluated different count addition schemes on the performance of cell clustering and projection after dimension reduction. By default, various methods were compared against log-normalization, which was used as the baseline for comparison. Thus, difference in performance in other methods are shown as gain or loss over the log-normalization, the method commonly used.

### CoDA LR-based transformations improve clustering performance

**Fig. 4:**
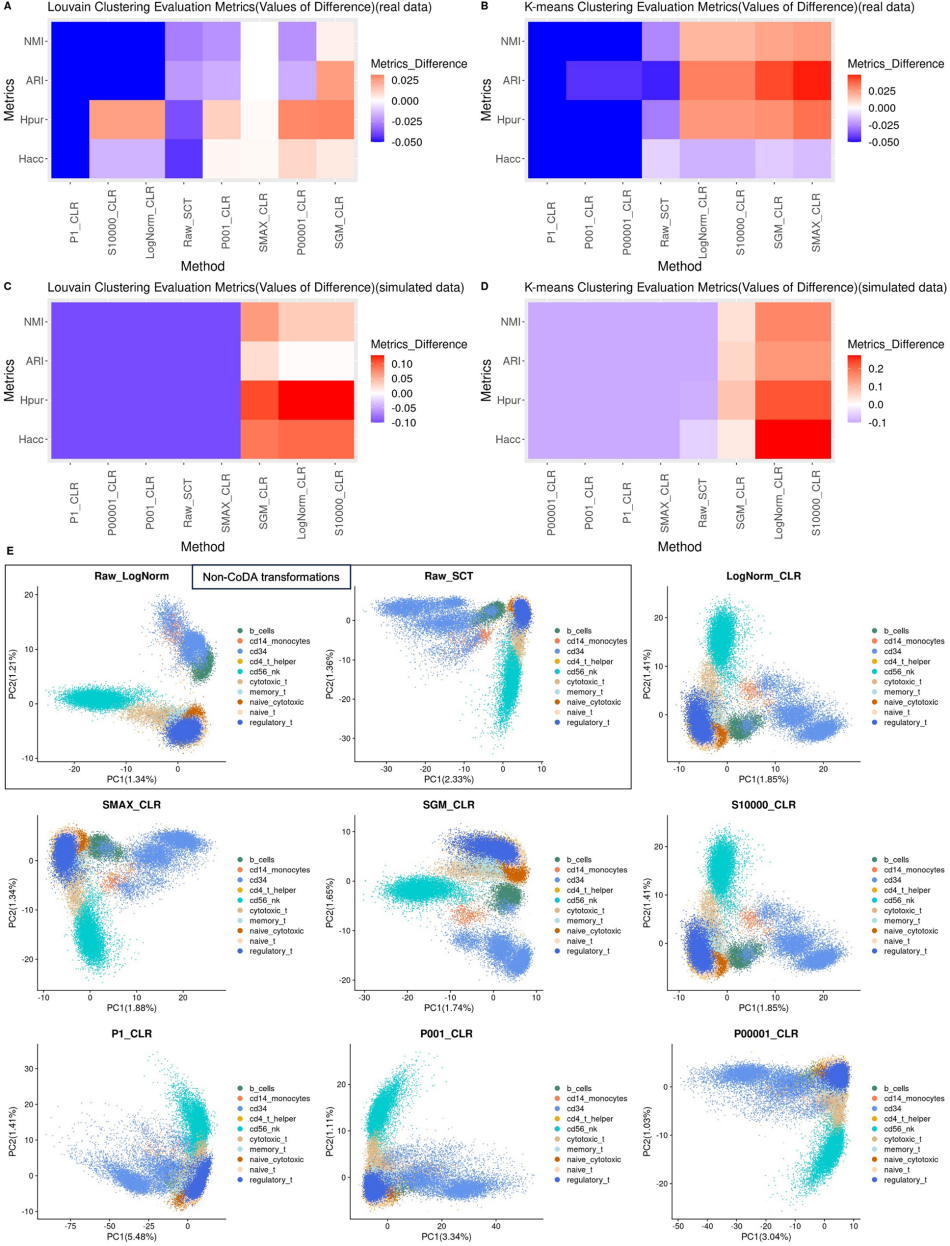
Handling of zero counts: clustering performance of different count addition schemes in CoDA transformations using K-means and Louvain Clustering algorithms on real and simulated datasets and the first 2-D PCA plots using the sorted PBMC dataset. Four evaluation metrics of clustering performance, Entropy of accuracy (${H}_{acc}$), Entropy of purity (${H}_{pur}$), Adjusted Rand Index (ARI) and Normalized mutual information (NMI) are used to quantitatively evaluate the clustering performance of different CoDA count addition schemes by Louvain Clustering and K-means Clustering. Four datasets with given cell-type labels (ground-truth) are used. The number of clusters is set to the number of known cell type labels in each dataset. Top 3000 high variable genes are used for running PCA. The top 10 PCs are used for clustering. ARI and NMI are subtracted by the results of the raw log-normalization (as baseline) (vice versa for ${H}_{acc}$ and ${H}_{pur}$ for better visualization) and the medians are taken. Extreme values in heatmaps have been limited to a cutoff for better visualization. CLR count addition schemes include: P1: plus 1; P001: plus 0.01; P00001: plus 0.0001; SMAX: plus Sum(j)/Max(Sum(j)); SGM: plus Sum(j)/Geometric-Mean(Sum(j)); S10000: plus Sum(j)/10,000; LogNorm_CLR: CLR transformed from log-normalized data. CLR transformation of log-normalized data (logNorm_CLR) produces better clustering than log-norm itself (Raw-LogNorm). Overall, those schemes adding a constant value (P1, P001, P00001) result in cell clusters that are less well demarcated. **A** and **B** Median metric difference values of the four real datasets using K-means and Louvain Clustering algorithms. **C** and **D** Median metric difference values of the four simulated datasets using K-means and Louvain Clustering algorithms. **E** First 2-D PCA plots of different CoDA count additions using the cell type sorted PBMC dataset

**Fig. 5:**
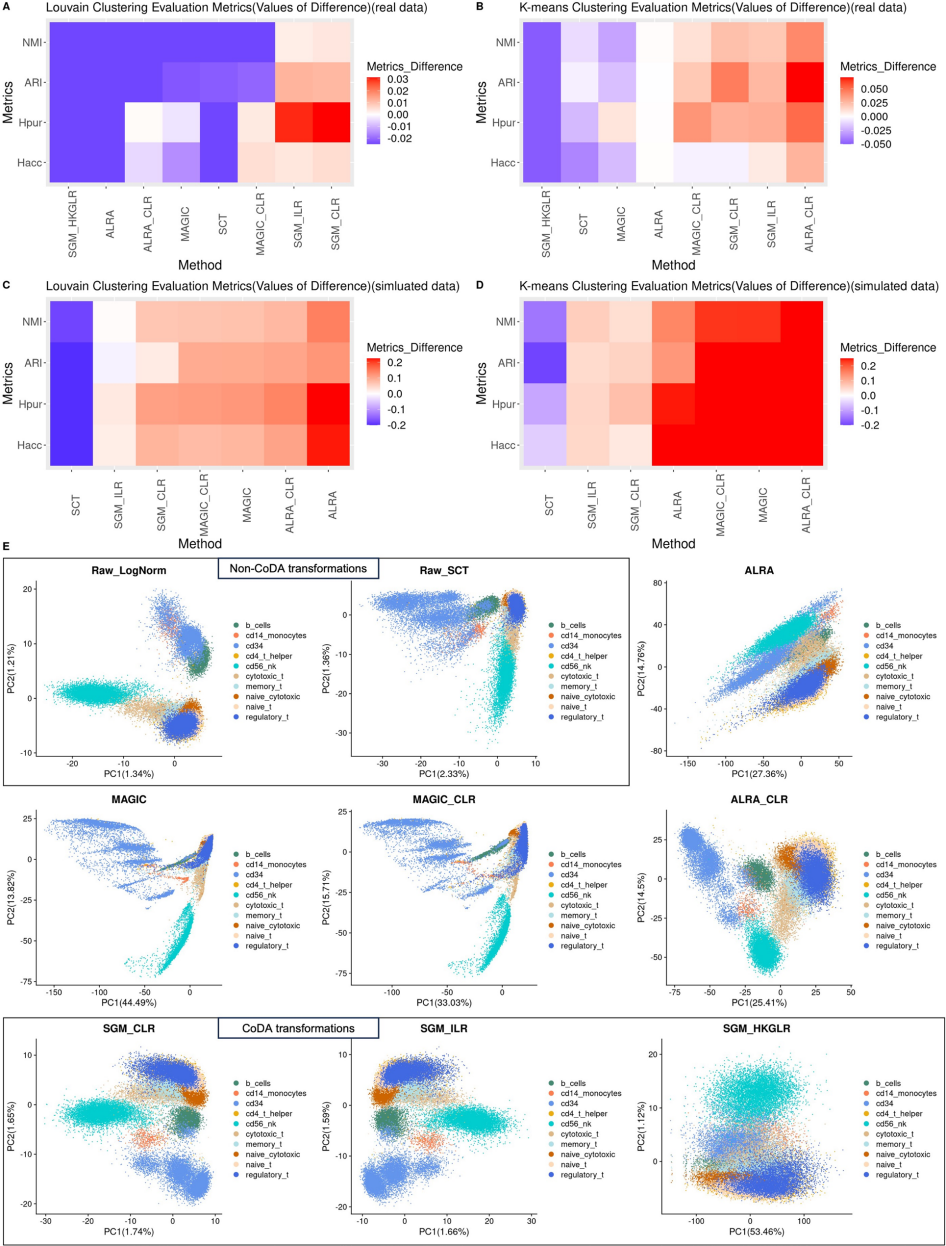
Clustering performance of different normalization methods and CoDA LR transformations using K-means and Louvain Clustering algorithms on real and simulated datasets and the first 2-D PCA plots using the sorted PBMC dataset. Four evaluation metrics of clustering performance, Entropy of accuracy (${H}_{acc}$), Entropy of purity (${H}_{pur}$), Adjusted Rand Index (ARI) and Normalized mutual information (NMI) are used to quantitatively evaluate the clustering performance of different normalizations/CoDA transformations/imputation algorithms by Louvain Clustering and K-means Clustering. Four datasets with given cell-type labels (ground-truth) are used. The number of clusters is set to the number of known cell type labels in each dataset. Top 3000 high variable genes are used for running PCA. The top 10 PCs are used for clustering. ARI and NMI are subtracted by the results of the raw log-normalization (as baseline) (vice versa for ${H}_{acc}$ and ${H}_{pur}$ for better visualization) and the medians are taken. Extreme values in heatmaps have been limited to a cutoff for better visualization. SGM_CLR represents the CLR transformation with count addition ‘Sum(j)/Geometric-Mean(Sum(j))’. Imputation algorithms produce extra spurious correlation between PC1 and PC2 (e.g., ALRA, MAGIC) which are not found in the datasets. Overall, CLR transformation of imputed data shows better clustering except in the simulated dataset undergone Louvain clustering. **A** and **B** Median metric difference values of the four real datasets using K-means and Louvain Clustering algorithms. **C** and **D** Median metric difference values of the four simulated datasets using K-means and Louvain Clustering algorithms. **E** First 2-D PCA plots of different normalizations/CoDA transformations/imputations using the sorted PBMC dataset

Quantitative evaluations of clustering were performed using four real datasets and four simulated datasets to compare various normalizations and LR transformations. In Fig. 4, we evaluated the effect of applying CLR transformation to various count addition schemes, while Fig. 5 compares the performance of various CoDA LR transformations–namely CLR, ILR and HKGLR, and other kinds of methods. CoDA LR transformations, particularly CLR using SGM count addition, showed great improvements in all clustering metrics (H_acc_, H_pur_, ARI, NMI) over conventional normalizations using either Louvain or K-means algorithms, across both real and simulated datasets (Figs. 4–5 and S3-4). CLR (LogNorm/S10000) also performed well in these evaluations. In contrast, constant count addition schemes (i.e., P1-CLR, P0.01-CLR, P0.0001-CLR) performed substantially worse. This confirms our hypothesis that cell-specific count addition based on total counts is more effective than simple constant addition and is also a prerequisite for successful CoDA-hd. Importantly, we demonstrated that log-normalized data can be directly transformed into CoDA LR representation via CLR (labelled as LogNorm-CLR in figures). This provides a straightforward way to apply CoDA to pre-normalized LogNorm datasets, which are commonly available in public repositories. Thus, the benefits of CoDA-hd are readily applicable to most public datasets.

In contrast, imputation by ALRA or MAGIC alone (labelled as MAGIC and ALRA in Fig. 5) did not perform well for real datasets. However, applying CoDA LR transformations to these imputed matrices greatly improve performance (comparing MAGIC-CLR vs MAGIC or ALRA-CLR vs ALRA). This suggests that the CoDA framework enhances the utility of imputation approaches.

Using simulated datasets, we also compared the metrics of various normalizations and count-addition CLRs against their ground truth (no dropout) datasets (i.e., True-LogNorm and True-CLR). As expected, True-SGM-CLR and True-LogNorm-CLR consistently outperformed True-LogNorm (Fig. S5). Since SGM-CLR emerged as the optimal count addition scheme, it was consistently used in other downstream analyses.

### CoDA LR transformations improve projections and visualizations of scRNA-seq datasets with known cell-type identities

In PCA and UMAP visualizations, CLR (SGM) and CLR (LogNorm/S10000) produced more discrete and biologically coherent cell-type clusters (Figs. 4 and S6). Compared to LogNorm alone, cell-types, especially monocytes, were better separated using CLR (Fig. 5). In contrast, knowledge-based denominator selection (HKGLR) performed poorly, generating complex and potentially misleading structures. Notably, CoDA revealed that certain cell-type relationships appeared more biologically plausible with CLR. For example, in UMAP projection of the PBMC data, naïve cytotoxic cells appeared distant from cytotoxic cells under log-normalization, whereas CLR appropriately positioned them closer, reflecting their biological relationship (Fig. S7).

Among zero-imputation methods, MAGIC and ALRA produced spurious, overly complex cell-type structures in PCA, which were absent in log-normalization or CLR. Interestingly, ALRA processed with CoDA LR transformations provided clearer cell-type projections in PCA (Fig. 5E).

Similarly, in the CellBench-10X-5CL dataset (Figs. S8–11), cell types were easily clustered and projected in PCA/UMAP across most normalizations/CoDA LR transformations. CLR (SGM) consistently performed well in these visualizations. In UMAP, H1975 cell lines formed separated clusters, suggesting subclones division (Fig. S11). Therefore, this dataset was selected for zero-inflation (degraded cells) spike-in experiment. Again, MAGIC generated most farther separated sub-clusters of the H1975 cell line which may represent an artefact due to imputation.

In simulated dataset 2, we included ground truth (no dropout) baselines: True-LogNorm and True-CLR.. Interestingly, in 2-D PCA, while most methods (including ground truth log-normalization) showed mixed cells without clear patterns, True-CLR and ALRA-CLR achieved better cluster separation than their log-normalized counterparts (Fig. S12). This again highlighted the advantage of CLR over the log-normalization. In 2-D UMAP, True-LogNorm and True-CLR produced distinct and compact cell-type clusters (Fig. S13). For the dropout dataset, CLR produced more distinct and concentrated clusters compared to log-normalization and were more similar to the ground truth.

### CoDA provides better clustering for degraded cells

**Fig. 6:**
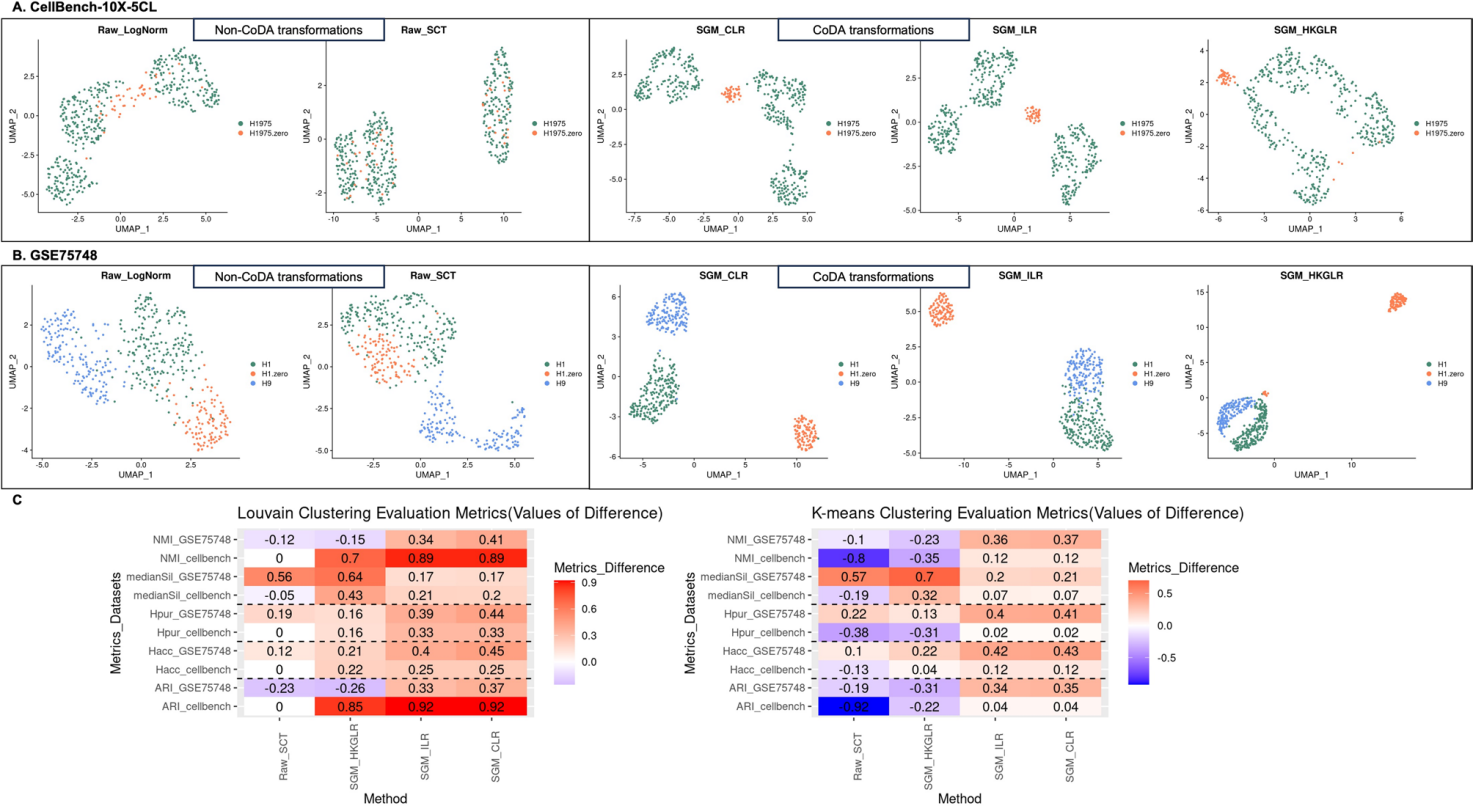
Effect of degraded cells: performances of different normalization methods and CoDA LR transformations when degraded cells are present. **A** 2-D UMAP plots of the CellBench-10X-5CL H1975 and H1975-zero subset processed by different normalizations and CoDA transformations. For the dataset CellBench-10X-5CL, 10% of the H1975 cells are randomly selected and copied to simulate low-quality cells. For each copied cell, 60% of the genes (~ 6000 genes) are randomly assigned to zero and then spike-in the data matrix as additional cells. Thus, the new data matrix has 10% more degraded H1975 cells with at least 60% feature (genes) counts set to zero. The data is then processed by various normalizations and CoDA LR transformations, followed by the evaluations on PCA and UMAP. For UMAP visualization, top 10 PCs are used. UMAP plots for H1975 cell subset are generated. Additionally, clustering performances are evaluated based on the separation of H1975 cells and the degraded cells (labeled as H1975-zero in the figure). **B** 2-D UMAP plots of the GSE75748-CellType H1, H1-zero, and H9 cell subset. Similarly, 40% of the H1 cells from dataset GSE75748-CellType are copied and simulated as spike-in degraded cells as described above. The separation of H1, H1-zero, and H9 cell clusters are then evaluated. **C** Results of the clustering performances of various normalizations/CoDA transformations on the two degraded cell datasets using Louvain and K-means algorithms. ARI and NMI are subtracted by the results of the raw log-normalization (as baseline) (vice versa for ${H}_{acc}$ and ${H}_{pur}$ for better visualization)

Since low-quality cells with high dropout rates may lead to misleading conclusions, we performed a simulation analysis by spiking degraded cells (10% of copied H1975 cells with zero-inflated parts) into the high-quality dataset CellBench-10X-5CL (see Methods). We studied whether CoDA LR transformations could improve clustering of these degraded zero-inflated cells which is essential to avoid erroneous results. In both 2-D PCA and UMAP visualizations, H1975-copied-zero cells (orange dots in Figs. 6A and S14A) were separated from original H1975 cells to varying degrees across methods, except in SC-Transform results (Fig. 6A). However, only CLR and ILR distinctly clustered these artificially degraded cells which is a desirable feature. In contrast, log-normalization scattered these degraded cells among distinct cell-type clusters, potentially misrepresenting them as transitional states. CLR, however, clearly grouped them as a separate low-quality cluster, suggesting that CoDA may help prevent misinterpreting technical artifacts as biological transitions. These results indicate that low-quality cells with high dropout rate can raise false trajectories between clusters, and CoDA may be a better alternative than conventional log-normalization in handling scRNA-seq data. In HKGLR UMAP, the degraded cells were spuriously split into two clusters and all cells were heavily scattered, which was repeatedly observed in different spike-in runs. Therefore, the use of knowledge-based denominator selection for CoDA transformations of scRNA-seq is not supported.

Similarly, in the GSE75748-CellType dataset, CoDA LR transformations better clustered the H1, H9 and degraded H1-copied-zero cells compared to conventional methods (Figs. 6B and S14B).

The quantitative evaluations confirmed that CLR showed improvements across all clustering metrics for both two subset zero-inflated datasets (Fig. 6C). This robustness to dropout effects represents a major advantage of CoDA for scRNA-seq analysis.

### CoDA improves trajectory inference

**Fig. 7:**
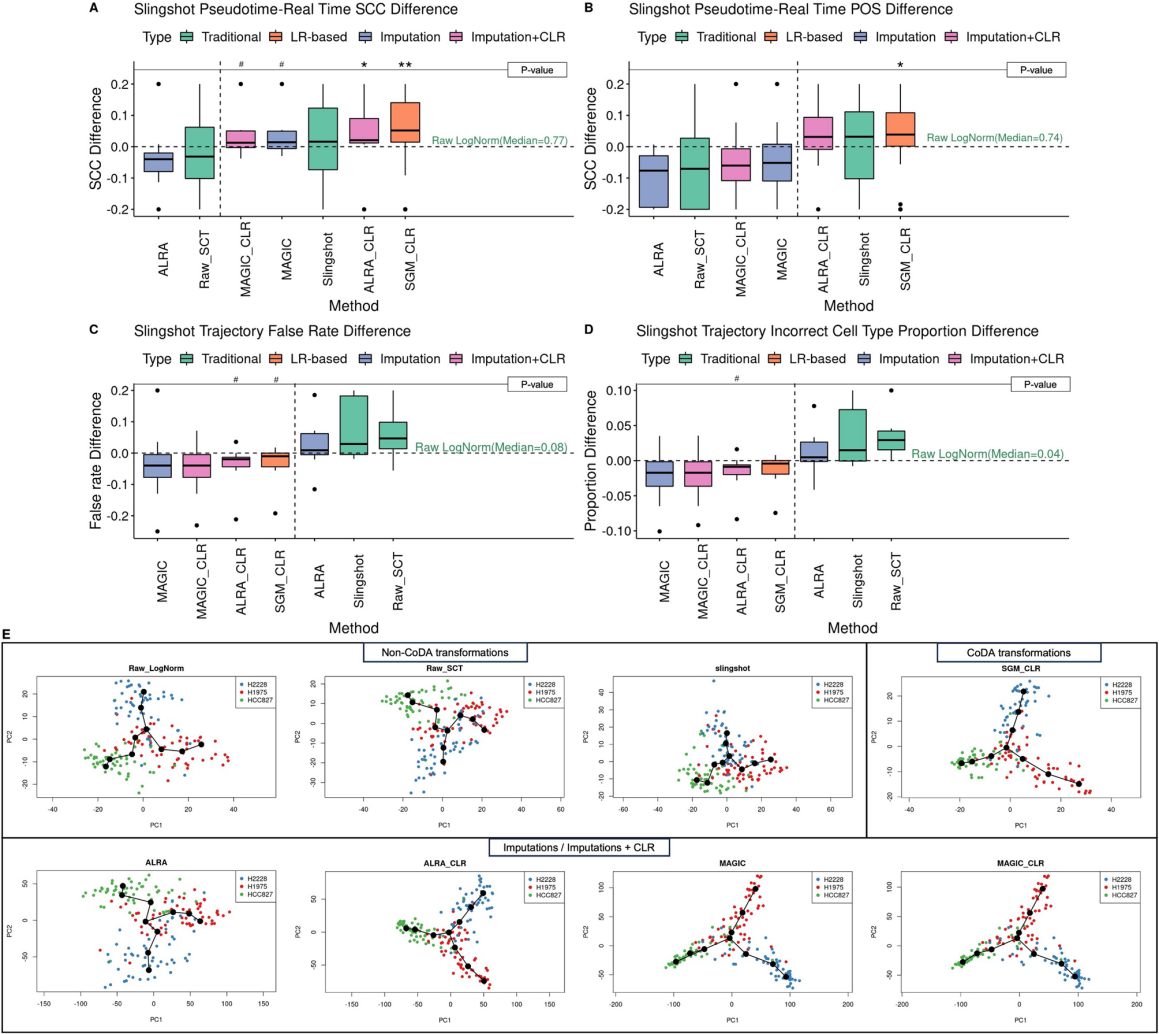
Comparison of effects of various normalizations/CoDA transformations on Slingshot trajectory inference. The performances of different normalizations/imputations/CoDA transformations on trajectory inference are evaluated with Slingshot using twenty-two datasets with real time & lineage (ground-truth) labels. The starting point of the trajectory is set based on known information and other parameters are set to default. For trajectory with multiple branches, the predicted branches and the real branches are matched by their largest overlaps of cells (see Methods). *, ** and *** represent ‘*P* < 0.05’, ‘*P* < 0.01’ and ‘*P* < 0.005’ respectively and ‘#’ stands for not-significant-difference (‘*P* < 0.1’), when compared to the baseline method raw log-normalization using Wilcoxon rank sum test. **A** and **B** Spearman correlation coefficients and Pseudo-temporal ordering scores are calculated between the predicted pseudotime and the real time labels. ALRA-CLR and SGM-CLR show significant improvement over baseline and Slingshot-default-transformation. **C** and **D** Generation of false results analyzed by using the trajectory false rates and the incorrect cell type proportions. Three CLR schemes in addition to MAGIC may have better (not statistically significant) control of false results generation. **E** The 2-D Slingshot PCA plots generated for the CellBench-10X cellmix3 dataset after different normalizations/imputations/CoDA transformations to visualize trajectories

When evaluating trajectory inference across twenty-two real gold standard datasets with known time labels, CLR showed statistically significant improvements in both Spearman correlation coefficients (SCC) (i.e., between inferred pseudotime and known time) and Pseudo-temporal ordering score (POS), compared to log-normalization, using Slingshot (Figs. 7A and B and S15). Interestingly, ALRA-CLR showed a substantial improvement compared to ALRA alone.

For datasets with multiple branches, CLR reduced false trajectory rates and decreased incorrect cell type proportions compared to log-normalization, using Slingshot (Figs. 7C-D and S15). 2-D visualization of trajectories (e.g., CellBench cellmix3, Fig. 7E) showed that CLR produced more compact and coherent trajectories, with cells from the same cell types appearing closer together and forming clearer continuums.

Similar improvements were observed with DPT, where CLR significantly enhanced trajectory inference (Fig. S16). In Monocle2, CLR consistently outperformed conventional normalizations and imputation methods (Fig. S17). However, in Monocle3, CLR did not improve performance but matched the consistency of log-normalization.

### Enhanced identification of biomarkers after CoDA LR transformation

**Fig. 8:**
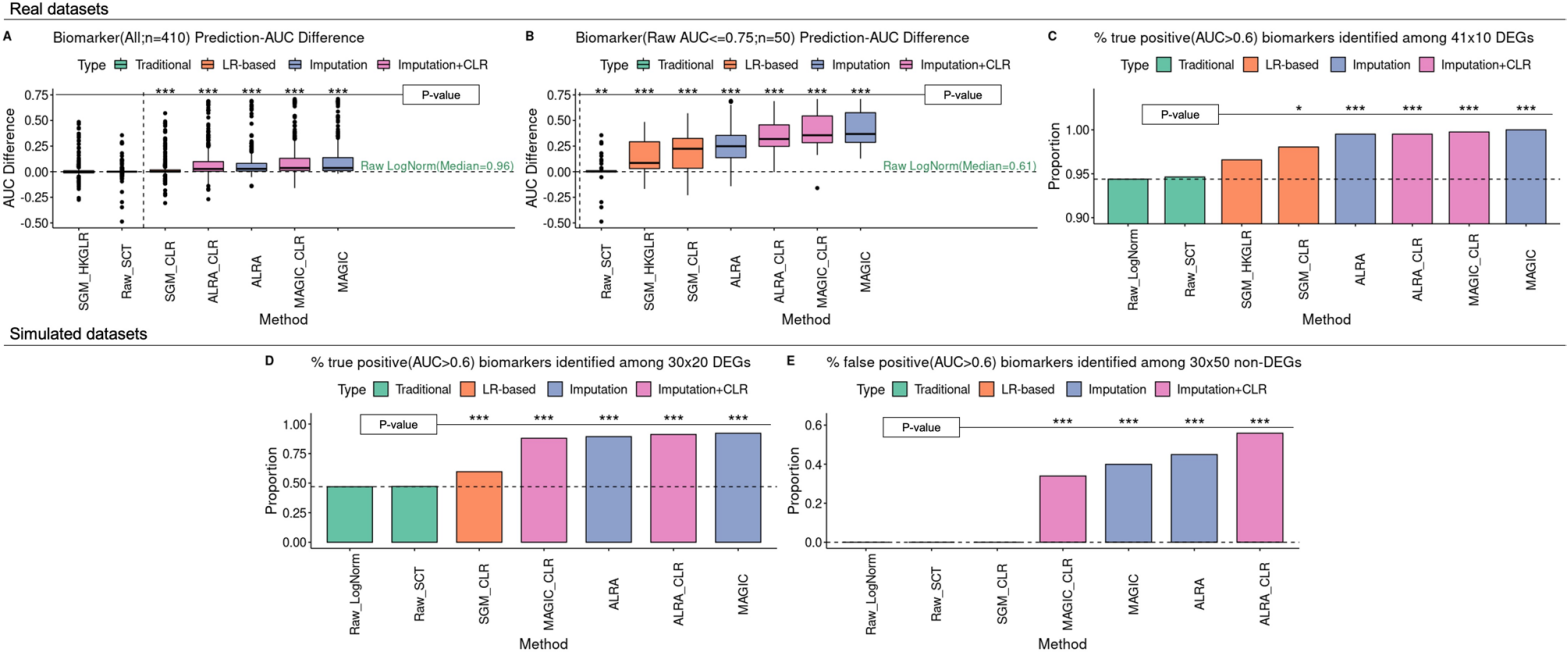
Performances of various normalizations/CoDA transformations on cell-type-specific biomarkers (DEGs) detection. The biomarker detection performances of different normalizations/imputations/CoDA transformations are evaluated. Top 10 DEGs between pairwise cell types in bulk data are used as real biomarkers in three real datasets (**A** and **B**). *, ** and *** represent ‘*P* < 0.05’, ‘*P* < 0.01’ and ‘*P* < 0.005’ respectively, when compared to the baseline method raw log-normalization using Wilcoxon rank sum test. **A** The AUCs for each method for each of the biomarkers are subtracted by the values of the raw log-normalization (baseline). AUCs of the biomarkers improve by CoDA transformations (SGM-CLR, MAGIC-CLR and ALRA-CLR) and imputation methods (ALRA, MAGIC). **B** When focusing on those DEGs that have only moderate AUC < = 0.75 on log-normalized data matrix, four CoDA transformations (SGM-HKGLR, SGM-CLR, ALRA-CLR, MAGIC-CLR) improve AUCs significantly. Both imputation methods also improve AUCs. **C** and **D** If AUC > 0.6 is used as cutoff for useful biomarkers, CoDA and imputation methods may enhance identification of biomarkers in real dataset (**C**) and simulation dataset (**D**). **E** However, in the simulation dataset with ground-truth, false positive biomarkers are only under control by Raw-LogNorm (as baseline), Raw-SCT and SGM-CLR. 30% to 50% of the selected non-DEGs are identified as potential biomarkers by ALRA or MAGIC (even with CLR transformation)

In the evaluation of biomarker identification, imputation methods (MAGIC, ALRA and their CLR-transformations) consistently improved true positive rate (Fig. 8). CLR showed modest improvements when considering all differentially expressed genes (DEGs) (Fig. 8A) but demonstrated significantly higher AUC values for markers that performed poorly with log-normalization (LogNorm AUC < 0.75 (Fig. 8B)).

Both LR transformations and imputation methods resulted in higher percentages of markers with AUC values greater than 0.6 (Fig. 8C and D), 0.75 or 0.9 (data not shown). This suggests that CoDA can enhance the performance of challenging biomarkers that conventional normalization struggles to identify.

Importantly, while imputation methods also increased the false positive rate (higher AUC for known non-markers (non-DEGs) in simulated dataset), CoDA LR transformations maintained specificity similar to conventional normalization (Fig. 8E). This balance of improved sensitivity without increased false positives represents another advantage of CoDA.

### Running time and memory usage

**Fig. 9:**
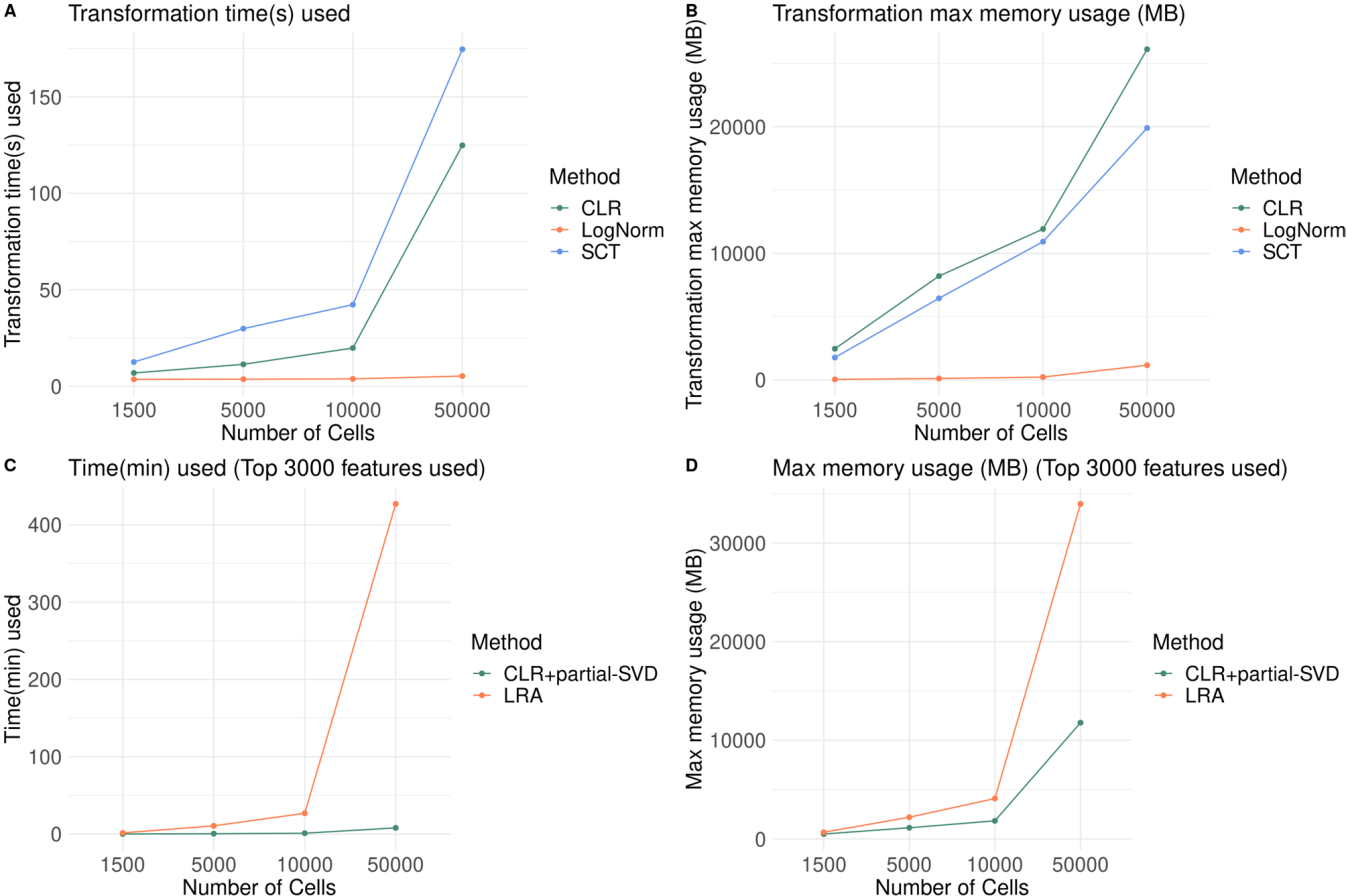
Computer resources (running time and memory requirement) needed for CoDA transformation (CLR) and analysis (CLR-partial-SVD and LRA). **A** and **B**: Running time (**A**) and memory requirement (**B**) for conventional log-normalization, SC-Transform and CLR transformation are compared. All of them are dependent on the number of cells in the data matrix. Four sizes of datasets (10,000 × 1500, 10,000 × 5000, 10,000 × 10,000 and 10,000 × 50,000 matrix) are applied. For the largest data matrix (10,000 × 50,000 matrix), CLR transformation can be completed within minutes using 20 GB RAM. **C** and **D**: Running time (**C**) and memory requirement (**D**) for CLR + partial-SVD and LRA (easyCODA) are compared. Time required (**C**) and memory usage (**D**) for performing CLR-partial-SVD are much smaller than conventional LRA in the easyCODA package

As expected, log-normalization was the fastest method regardless of cell number, while SC-Transform was the slowest. CLR required more computational resources than log-normalization but less than SC-Transform (Fig. 9A and B). For dimension reduction, our ‘CLR + partial-SVD’ approach was substantially faster and more memory-efficient than standard LRA implementations, making CoDA feasible even for large datasets (Fig. 9C and D). Both procedures completed within hours using up to 35 GB RAM which are typical setup in servers and even on desktop computer. Such computing power requirement enables CoDA-hd to be used by most researchers.

## Discussion

In this study, we successfully applied the complete CoDA workflow, from data closure and LR transformation to dimension reduction. These procedures are compatible with commonly used scRNA-seq downstream analysis algorithms. The two key innovations in our study are (1) SGM count addition scheme developed specifically for scRNA-seq (and potentially other sparse data matrices) and (2) CoDA of high dimensional data. Using more than thirty real or simulated datasets, we demonstrated some advantages of CoDA LR transformations, especially CLR with SGM count addition, in multiple downstream analyses of scRNA-seq.

We pioneered new count addition schemes (e.g., SGM count) and CLR after closure of data treated by log-normalization. These two CoDA techniques improved data representation of scRNA-seq data matrix. The use of partial SVD also allows PCA of LR to be finished seamlessly for high dimensional matrix in a matter of minutes, in contrast to the standard CoDA LRA algorithm which is designed for smaller datasets.

Our SGM count addition scheme overcomes the hurdle of using sparse data matrix in CoDA. It also preserves cell-specific characteristics which can be revealed on PCA or other embedding methods, and provided distinct and biologically coherent clustering patterns in dimension reduction visualizations. CLR shows many advantages and great compatibility with various count addition schemes.

One key benefit of CoDA is its superior handling of low-quality, zero-inflated cells. By correctly identifying degraded cells as separate clusters rather than transitional states, CoDA helps prevent misinterpretation of technical artifacts as biological phenomena. We recently showed that the degraded cells were a primary cause of artefactual results and conclusions from trajectory analysis of scRNA-seq [6]. Applying CoDA to re-analyze the data will help to avoid such mistakes and wrong conclusions.

CoDA-hd is practical for routine applications. Although CoDA requires more computational resources than log-normalization, the benefits in downstream analyses justify this trade-off. Furthermore, our implementation of fast truncated SVD for dimension reduction keeps CoDA practical even for large datasets. As many public datasets have been prior normalized (typically via log-normalization), we demonstrated that log-normalized data can be readily converted to CoDA via CLR. Therefore, many public datasets can take advantage of CoDA.

It is interesting to note that CoDA LR transformations like CLR can be helpful for correct trajectory analysis. This improvement suggested that gene ratios can be powerful indicators of continuous cell-state transitions and cell–cell relationship. In biomarker prediction, biomarkers with low predictive abilities using log-normalization had much better performance when using CoDA LR transformations. The gene ratios again demonstrated their potentials to be used for classifications and clinical diagnosis.

We here demonstrate that CoDA delivers outstanding performance with specific count addition scheme for sparse matrices like scRNA-seq. This approach could also be applied to other high dimensional sparse matrices of big data in other disciplines, such as those in business analytics.

This study has several limitations. First, due to the limited computational resources and scalability considerations, our analysis was restricted to datasets comprising up to approximately 50,000 cells that had also been used in previous benchmark studies. For clustering evaluation, we exclusively used datasets with single ground-truth cell-type label, as the adopted metrics require definitive true labels for calculation. Consequently, datasets with complex experimental designs or multiple valid label schemes (e.g., cell types, experimental conditions, donor information) were excluded to reduce complication in our clustering assessments. Future studies could focus on incorporating larger-scale datasets for more comprehensive validation. Second, our comparative analyses were conducted using common and well-established methods (e.g., K-means clustering, Slingshot trajectory analysis, AUC evaluation) due to their universal applicability across different data normalization approaches. While this ensures fair comparisons by avoiding specialized tools designed for particular data types, we acknowledge that method-specific approaches (e.g., MAST hurdle model, Leiden clustering, or alternative pseudotime inference algorithms) might provide additional insights when evaluating optimal analytical strategies. Third, our evaluations only focused on core downstream analytical tasks. Given the expanding downstream applications of scRNA-seq, e.g., cell–cell communication analysis and SCENIC regulatory analysis, future investigations could assess CoDA's compatibility with these advanced analytical frameworks. Finally, we recognize certain inherent limitations of the CoDA approach itself. The CoDA LR transformation process may demand greater computational resources (both in processing time and memory allocation) compared to conventional log-normalization. However, this is still acceptable for typical single-cell datasets when utilizing adequate computational resources. For exceptionally large datasets, an easy and practical solution is to conduct data partitioning.

## Conclusions

The compositional nature of scRNA-seq data has been long neglected due to various reasons, like its sparse nature and lack of CoDA software. But the fact that scRNA-seq data is compositional cannot be denied. Here, we proposed theoretically sound and practical methods and associated framework to analyze high dimensional data by CoDA. By treating scRNA-seq data by CoDA, we gain certain advantages in cell clustering and visualization, handling of low-quality cells, trajectory inference, and biomarker identification.

## Supplementary Information

Below is the link to the electronic supplementary material.

Supplementary Material 1

## Data Availability

Public datasets used in this study were summarized in Table S3. Some example datasets were also placed in: https://github.com/GO3295/CoDAhd. The code for the evaluations and implementing CoDA LR transformations (R package ‘CoDAhd’) was placed at https://github.com/GO3295/CoDAhd.

## Abbreviations

CoDA: Compositional data analysis
scRNA-seq: Single cell RNA-seq
LR: Log ratio
CLR: Centered-log-ratio
ILR: Isometric-log-ratio
TA: Transcript abundances
PCA: Principal component analysis
UMAP: Uniform manifold approximation and projection
HTS: High-throughput sequencing
DEG: Differential expressed gene
ALR: Additive-log-ratio
HKGLR: Housekeeping-gene-log-ratio
LRA: Log-ratio analysis
SVD: Singular value decomposition
ARI: Adjusted rand index
NMI: Normalized mutual information
PBMC: Peripheral blood mononuclear cells
SCC: Spearman correlation coefficients
POS: Pseudo-temporal ordering score
AUROC: Area under the receiver operating characteristic curve
FC: Fold changes
